# Long-term outcomes of “open iridectomy” for secondary anterior chamber epithelial iris cysts

**DOI:** 10.1038/s41598-021-96012-4

**Published:** 2021-08-16

**Authors:** Jie Lan, Ting Liu, Yusen Huang, Xiaojing Pan, Yufen Wei, Pingzhong Xie, Qianqian Kong, Xiang Guo, Lixin Xie

**Affiliations:** 1 Qingdao Eye Hospital of Shandong First Medical University, Qingdao, China; 2 State Key Laboratory Cultivation Base, Shandong Provincial Key Laboratory of Ophthalmology, Shandong Eye Institute, Shandong First Medical University and Shandong Academy of Medical Sciences, 5 Yanerdao Road, Qingdao, 266071 China

**Keywords:** Eye abnormalities, Pathogenesis

## Abstract

Epithelial cysts run a high risk of recurrence and conversion to sheet-like ingrowth after surgical intervention. In this retrospective study, we introduced a modified iridectomy for treatment of secondary epithelial iris cysts (EICs) in the anterior chamber. Twenty-nine patients (29 eyes) aged 2–61 years received “open iridectomy” for EICs between April 1995 and July 2019. After viscodissection, most of the cyst wall was cut using a 20-gauge aspiration cutter via a 2.5-mm clear corneal incision. The residue closely adhering to the iris stroma was remained to avoid photophobia and diplopia. At 3 months, best corrected visual acuity was ≥ 20/100 in 55.5% (15/27, except two pediatric patients with poor cooperation) of patients. Among the eight patients suffering partial corneal edema preoperatively, six patients received surgery treatment at 3–6.5 months, and the cornea in the other two patients became transparent after medication. In a mean follow-up of 47.4 months, recurrence occurred in 3 patients at 7, 37, and 118 months, respectively. The percentage of treatment success was 96%, 87%, and 65% at 1, 5, and 10 years, respectively. “Open iridectomy” was effective for EICs, with a minimal invasion, less damage to the corneal endothelium, and a low recurrence rate.

## Introduction

Anterior chamber iris cysts can be congenital or acquired as a complication of penetrating trauma or intraocular surgery. Congenital cysts arise endogenously and usually keep stable without clinical symptoms, while secondary epithelial iris cysts (EICs) might require surgical intervention and have a poor prognosis for high risks of recurrence and conversion to sheet-like ingrowth. Conservative therapies for EICs, including Nd:YAG laser cystostomy^1–3^, needle aspiration alone^4^ or combined with photocoagulation^5,6^ or endodiathermy^7^, and intracystic irrigation^8–10^, may lead to uveitis, secondary glaucoma, and recurrence^11,12^. Aggressive surgical excision and devitalization of the epithelial tissues with vitrectomy, lensectomy or even full-thickness corneoscleral grafting^13,14^ can damage ocular structures and also increase the risk of recurrence^15,16^.

By far there have been only a few case reports about the management of secondary EICs with a short follow-up, possibly due to the low morbidity of the disease. In this study, we described a modified surgical approach named “open iridectomy” which we used for clinical treatment of EICs for over 20 years and evaluated its long-term therapeutic results with a comparatively large cohort.

## Methods

### Subjects

Medical records of a consecutive series of 29 patients (29 eyes), 19 males and 10 females, who received surgical treatment for secondary EICs between April 1, 1995 and July 31, 2019, were retrospectively reviewed. This study was approved by the Institutional Review Board of Qingdao Eye Hospital. Informed consent was obtained from the patients involved.

The patient age at surgery ranged from 2 to 61 years (mean, 30.4 ± 17.2 years). The mean follow-up was 47.4 ± 46.9 months (range 5–180 months). The cysts were secondary to penetrating trauma in 26 eyes (89.7%) and cataract extraction in 3 eyes (10.3%). One of the eyes had a recurrent EIC after receiving the first treatment in a local hospital. Visual axis obstruction (25/29, 86.2%) was the predominant concurrent complication, followed by cataract (9/29, 31.0%), corneal edema (8/29, 27.5%), glaucoma (2/29, 6.8%), and lens dislocation (1/29, 3.4%).

All cyst extractions were performed by the same surgeon (LX). Inclusion criteria were: (1) enlarged cysts obstructing the visual axis, (2) ultrasound biomicroscopy (UBM) or anterior segment optical coherence tomography (As-OCT) showing cyst attachment to the anterior chamber angle and corneal endothelium, possibly accompanied with partial corneal edema, (3) repeated iridocyclitis, secondary glaucoma, complicated cataract, or lens subluxation, and (4) cysts in the anterior chamber allowing a transcorneal approach.

### The “open iridectomy” technique

**Figure 1:**
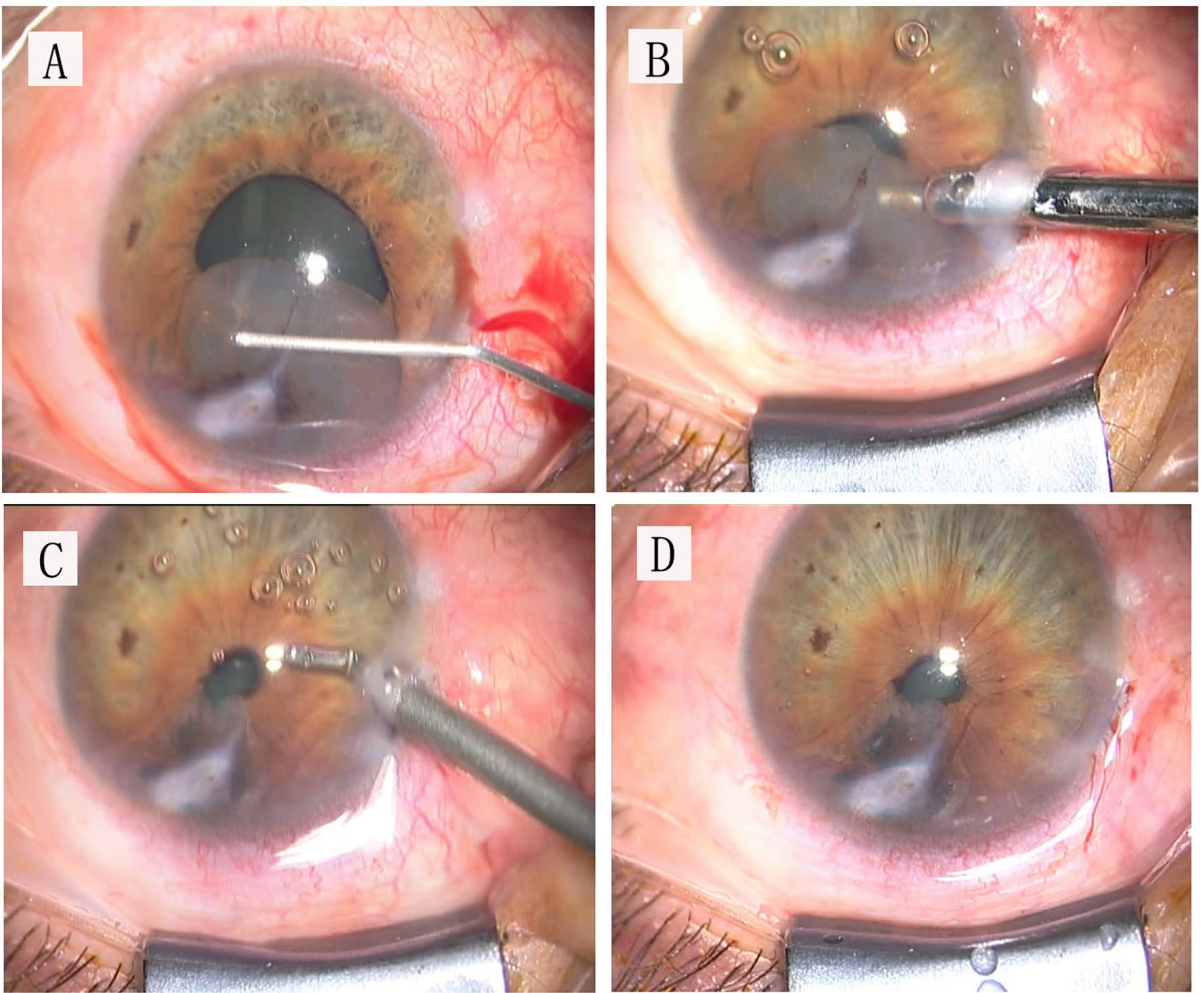
The main steps of “open iridectomy”. (**A**) The cyst wall was viscodissected from the corneal endothelium and anterior chamber angle till to the cyst base. (**B**) A 20-gauge vitrector was introduced to aspirate and resect the cyst wall till to the cyst wall/base which could preserve most iris stroma. (**C**) The cystic fluid and viscoelastic substance were removed by irrigation and aspiration. (**D**) The incision was self-sealed.

Local or general anesthesia was performed according to the patient cooperation. A side incision was created for injection of carbachol 0.1 mg/ml (Bausch & Lomb, Jinan, China) to induce miosis and sodium hyaluronate 15 mg/ml (Qisheng, Shanghai, China) to maintain the depth of the anterior chamber. Then a 2.5-mm limbal incision was made, through which sodium hyaluronate 15 mg/ml was filled to separate the cyst wall from the adjacent intraocular structure (corneal endothelium and anterior chamber angles) till to the cyst base. Normal tissues were preserved when a 20-gauge aspiration cutter (DP4400-6, Bausch & Lomb, Rochester, New York, USA) was introduced to aspirate and resect most of the cystic content and collapse the cyst wall. The suction vacuum was set at 200 mmHg, the cutting rate at 800 cuts per minute (cpm), and the BSS bottle at 70 cm. Because of the perfusion during the aspiration cutting, an anterior chamber maintainer was not necessary. To avoid photophobia and diplopia related to excessive iridectomy, the residual cyst wall/base closely adhering to the iris stroma was remained. The cystic fluid and viscoelastic substance were removed by irrigation and aspiration (MVS1063C, Bausch & Lomb, Rochester, New York, USA) with a BSS bottle set at 70 cm through the limbal incision. The incision could be self-sealed or sutured with one stitch (Fig. 1). The resected walls of the iris cysts were histopathologically examined. The surgical procedure is shown in Supplementary video 1.

### Postoperative therapy

Postoperatively, all patients were administered with tobramycin 0.3% and dexamethasone 0.1% eyedrops (Alcon, Fort Worth, Texas, USA) four times a day for 2 weeks and pranoprofen eyedrops (Senju, Osaka, Japan) four times a day for 1 month. The follow-up visits were scheduled at 2 weeks, 1 month, 3 months, and as necessary thereafter.

### Outcome measures

Postoperative evaluation included visual acuity testing, intraocular pressure (IOP) measurement (Full Auto Tonometer TX-F, Canon, Tokyo, Japan), slit-lamp microscopy (BQ900, Haag-Streit AG, Koniz, Switzerland), UBM (SW-3200L, SUOER, Tianjin, China), As-OCT (Visante OCT, Carl Zeiss Meditec, Dublin, CA, USA), surgical complications, recurrence, and further surgical procedures.

### Statistical analysis

Statistical analysis was carried out with SPSS software version 17.0 (SPSS, Inc., Chicago, IL, USA). The age and follow-up time were described as mean ± standard deviation (SD) after Kolmogorov–Smirnov test. Kaplan–Meier analysis was used to assess the recurrence.

### Ethical approval

All procedures performed in studies involving human participants were in accordance with the ethical standards of the institutional and/or national research committee and with the 1964 Helsinki declaration and its later amendments or comparable ethical standards.

## Results

### Histopathological examination

**Figure 2:**
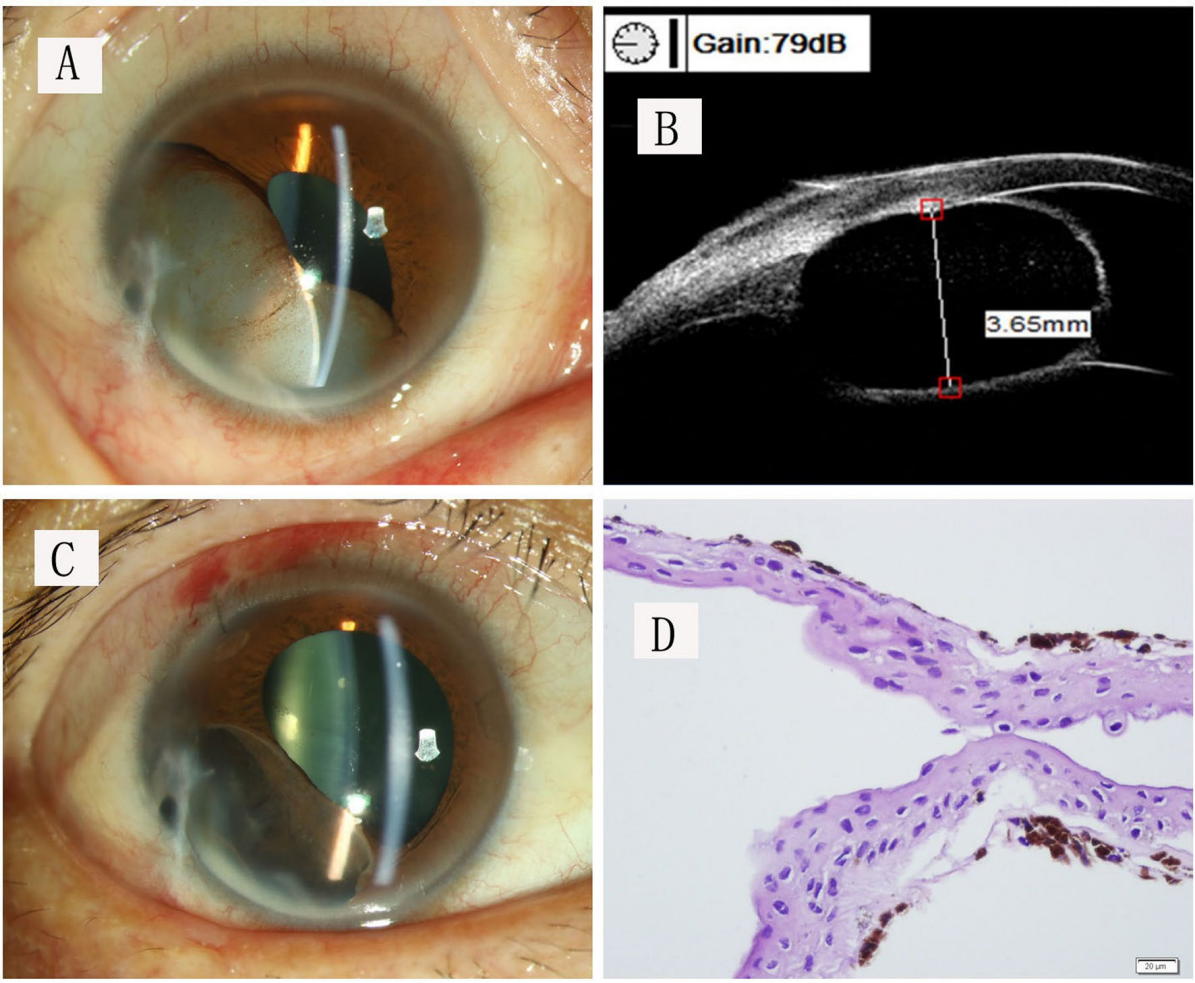
Treatment outcomes of this “open iridectomy” technique. (**A**) An iris cyst covered part of the pupil in the anterior chamber. (**B**) The UBM image showed the cyst attached to the anterior chamber angle and corneal endothelium. (**C**) The iris stroma and the pupil were preserved after the cyst wall was removed. (**D**) Pathological examination illustrated a non-keratinised stratified squamous epithelium (hematoxylin–eosin staining; bar = 20 microns).

Histopathological examination of the cyst tissue demonstrated iris stroma and pigment granules lining several layers of non-keratinised squamous epithelial cells (Fig. 2). The fluid aspirated from the cyst was also found to contain some epithelial cells. In the two patients with recurrence (patients 1 & 2 in Table 2), fewer than 3 layers of squamous epithelial cells were seen to be extensively adherent to the iris stroma.

### Visual outcome

Except two pediatric patients with poor compliance, the patients’ best corrected visual acuity (BCVA) ranged from hand motions to 20/30 at 3 months after surgery. The visual acuity was improved in 14 eyes (14/27, 51.9%), unchanged in 8 eyes (8/27, 29.6%), and declined in 5 eyes (5/27, 18.5%) for progressive corneal decompensation (preoperative existence) or posterior capsular opacification. Fifteen eyes (15/27, 55.6%) had the BCVA equal to or better than 20/100.

### Complications and postoperative therapy

During the modified iridectomy, nine of 29 (31.0%) patients received combined surgeries (Table 1). Further surgical interventions were required in 14 (48.2%) patients. In the 8 eyes with concurrent preoperative corneal edema, 6 eyes developed endothelial decompensation, which was treated by corneal surgeries.

**Table 1:** Surgical procedures and postoperative characteristics of patients with secondary epithelial iris cysts.

Parameters	Eye number	
**Combined surgery**		
Cataract extraction	8	
Trabeculectomy	1	
**Postoperative complications and managements**		
Endothelial decompensation	6	Penetrating keratoplasty (5), amnion transplantation (1)
Glaucoma	4	Trabeculectomy (2), transscleral diode laser cyclophotocoagulation (2)
Cataract	2	Cataract exaction
Posterior capsular opacification	1	YAG laser posterior capsulotomy
Sheet-like ingrowth	1	Scleral grafting

**Figure 3:**
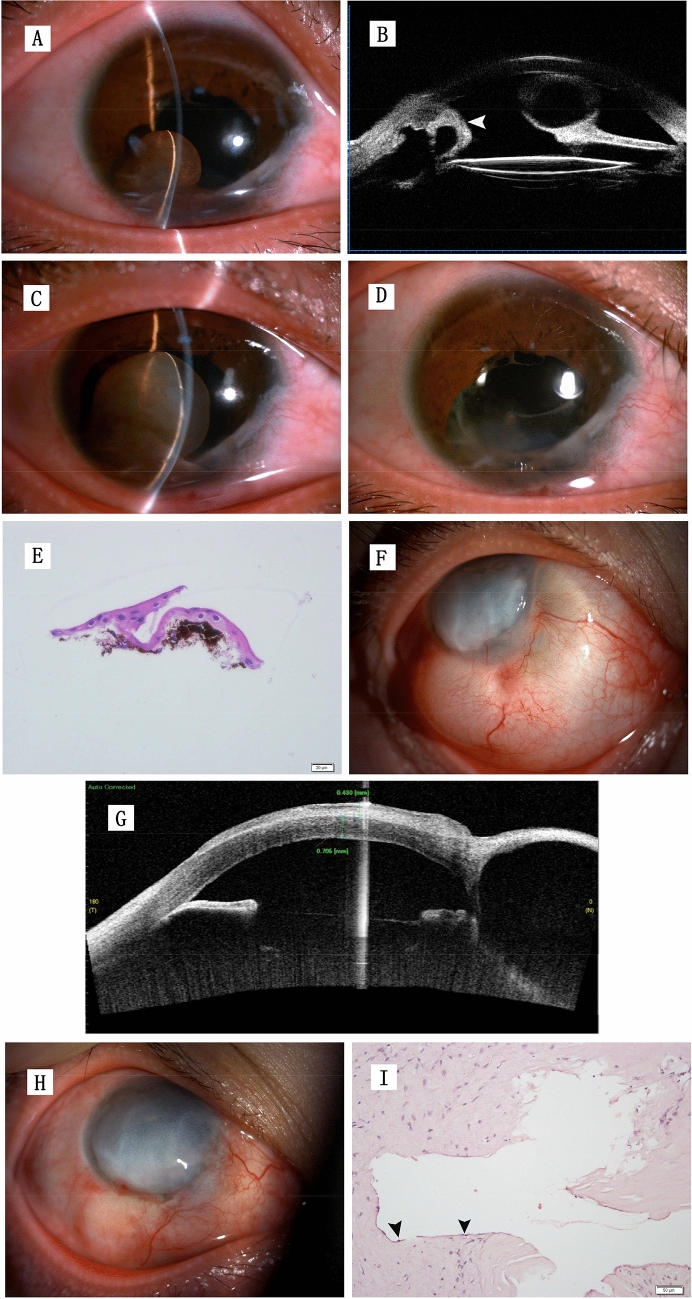
The therapeutic process of a 26-year-old female patient with an epithelial iris cyst and subsequent sheet-like ingrowth. (**A**) A recurrent cyst in the anterior chamber after congenital cataract surgery. (**B**) UBM showed the cyst in the iris and suspected sheet-like ingrowth in the opposite position (white arrow). (**C**) The cyst enlarged and contacted with the corneal endothelium after 5 months of follow-up. (**D**) Slit-lamp microscopic photograph after the cyst extraction. (**E**) A few stratified layers of the squamous epithelium with underlying pigment granules in the cyst wall (hematoxylin–eosin staining; bar = 20 microns). (**F**) Progressive sheet-like ingrowth was noticed in the nasal and inferior sides 4 years after the cyst extraction. (**G**) As-OCT demonstrated the sheet-like ingrowth was limited below the conjunctiva with no recurrence in the anterior chamber; cornea endothelial decompensation was noticed with the corneal thickness being 1130 μm. (**H**) Image at 10 months after epithelial sheet-like ingrowth resection and scleral transplantation. (**I**) Postoperative pathological results showed inflammatory hyperplasia of fibrous connective tissue; the black arrow indicates the inlayer cyst wall covered by a thin layer of the squamous epithelium (hematoxylin–eosin staining, bar = 50 microns).

One eye suffered sheet-like ingrowth after the cyst excision (Fig. 3). The eye had received congenital cataract surgery 20 years before and iris cyst extraction 2 years before in a local hospital. The patient first visited our institution in December 2013 for the recurrence of an EIC at the 7–8 o’clock positions. UBM showed suspected sheet-like ingrowth at the 4–5 o’clock positions. For the enlarged cyst, “open iridectomy” was performed in May 2014 with a histopathological examination. In May 2018, the progressive epithelial sheet-like ingrowth with corneal edema was resected, and scleral transplantation was performed. No recurrence or aggravated corneal edema was found during the 6 months of follow-up.

### Recurrence and treatment

**Figure 4:**
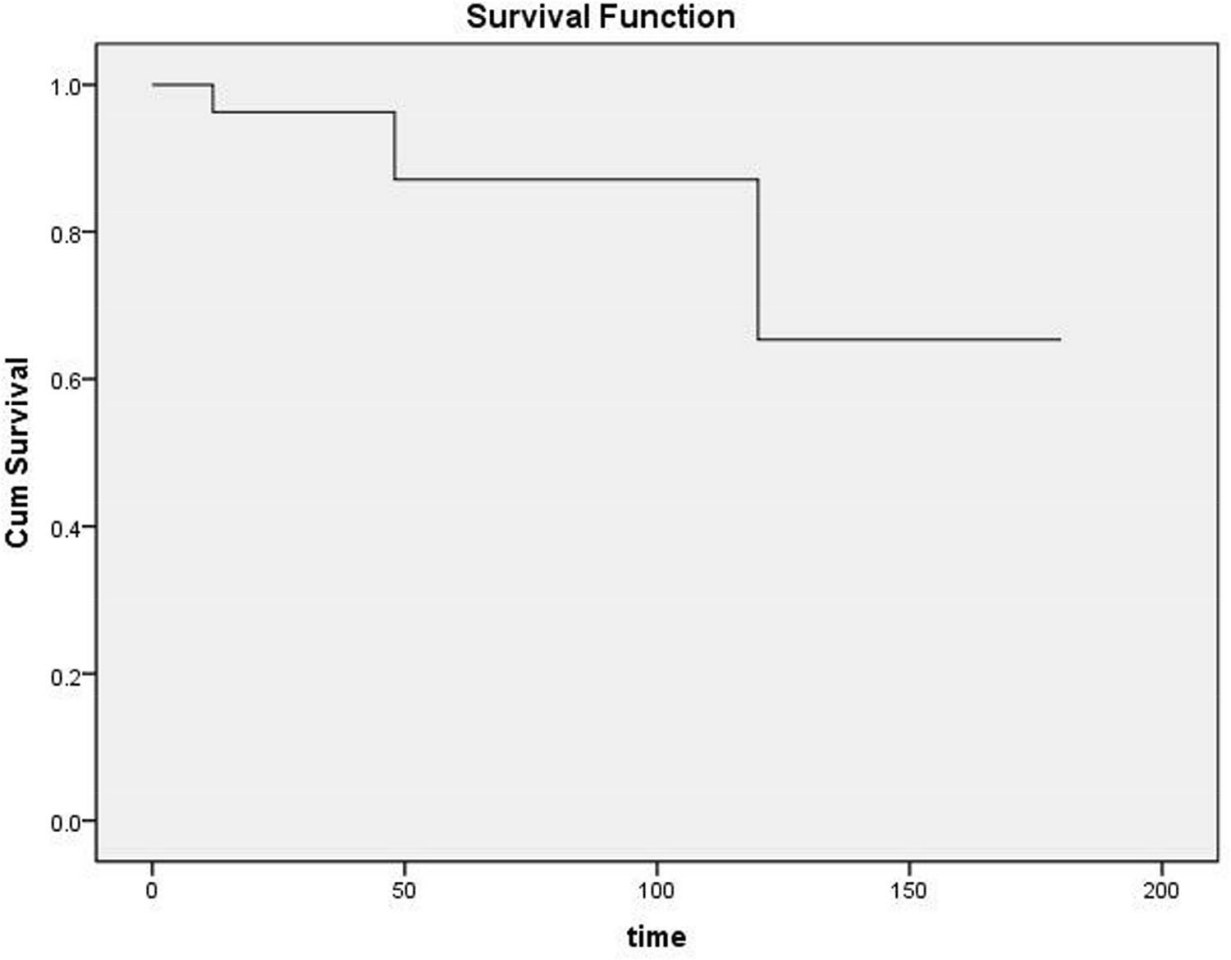
Kaplan–Meier survival curve of the cyst recurrence.

Repeated excisions were required in three eyes at 7, 37, and 118 months, respectively. The details of the cases are listed in Table 2. Except one patient (case 3) who received repeated excision combined with cataract extraction in another hospital, the other two patients received a second “open iridectomy”, with no recurrence observed during the follow-up of 19 and 27 months, respectively. The cumulative percentage of treatment success in all 29 patients was 96%, 87%, and 65% at 1, 5, and 10 years, respectively (Fig. 4).

**Table 2:** Details of the recurrence and management.

Patient	Cyst etiology	Age/sex	Combined procedure	Recurrence time (months)	Surgical treatment
1	Trauma	19 yrs/M	Cataract extraction	7	Open iridectomy
2	Trauma	61 yrs/F	No	118	Open iridectomy
3	Trauma	46 yrs/M	No	37	Cyst excision combined with cataract extraction^a^

*yrs, years; M, male; F, female; a, the surgery was performed at another hospital.

## Discussion

It is a challenge to strike a balance between minimally invasive surgery and a low recurrence rate for the management of secondary EICs. The “open iridectomy” technique we used was to open the cyst wall to the anterior chamber through a small incision with simple instruments. Most of the cyst wall and content were cleaned by an aspiration cutter, and the few production of the remained tissue was excreted by aqueous humor circulation. The open area was made large enough to avoid cyst reclosing, and the normal tissue could be protected by precision cutting. All patients in this study achieved favorable visual function recovery with a low recurrence rate during the mean follow-up of 4 years.

According to previous reports, non-invasive procedures by laser with a small open area and content cleaning just through the aqueous humor circulation may result in recurrence and sheet-like ingrowth^3,17^. Actually, we do observe rapid proliferation of cysts after laser treatment, and the recurrent cysts are liable to cause secondary complications like glaucoma, corneal edema, and structural changes in the anterior chamber over time. Cyst aspiration combined with endodiathermy^7^ or endolaser photocoagulation^6^ at the excision site for devitalization of residual cells has been tried in a small number of cases with a short period of follow-up. Injection of chemicals, such as ethalnol^8^, into the cyst requires a precise judgment of the cyst status by the surgeon. Once the procedure fails, the chemicals may be leaked and induce a disaster in the eyeball.

Invasive surgery, like EIC excision combined with corneal or corneoscleral grafting, has been used in treating progressive cystic or diffuse epithelial ingrowth of the anterior chamber^14,18^ as a permanent solution compared with conservative methods. Although the epithelial tissue could be completely removed to avoid recurrence, serious complications, including hemorrhage, secondary glaucoma, inflammation, and rejection of the graft tissue, may occur. The visual function is inevitably deteriorated because of the pupil defect and astigmatism.

Anterior chamber EICs, usually with delineated margins, can be visible during the surgical procedure. Hence we treated the cyst by lamellar iridectomy instead of sector iridectomy for prevention of glare and dicoria. Our modified technique possesses the following advantages in the treatment of secondary EICs. First, the 20-gauge instrument for vacuum aspiration could maintain a stable anterior chamber. The EIC is amenable via a localized, more conservative, and less destructive excision, which has the potential for commensurately less collateral damage to the delicate ocular structures and thus less visual disturbance. Second, a 2.5-mm incision allows combined surgical procedures, like phacoemulsification or trabeculectomy. Third, the procedure could be safely repeated.

In this study, there were 8 eyes with coexisting corneal edema before the excision surgery, 6 of which received penetrating keratoplasty or amniotic membrane transplantation months after the intervention, while 2 eyes had transparent corneas after medication, with no new onset of corneal decompensation during the follow-up. In the viscodissection of the cyst wall from the corneal endothelium, our approach did help to preserve the corneal endothelium and eliminate the corneal edema. Moreover, postoperative glaucoma was found in 4 eyes, reminding us that even if the cysts are successfully eradicated, IOP should be monitored for a long period of time.

The recurrence time of iris cysts was reported to range from 6 weeks to 5 years depending on different surgical treatment methods^19–21^. To avoid recurrence, Shields et al.^22^ advised to perform an aspiration and light cryotherapy, and recommended a non-vitrectomy technique to prevent the inflammatory reaction and epithelial downgrowth. Shanbhag and colleagues^14^, however, demonstrated that broad-based surgical iridectomy might completely clean up the retained cells in the iris. Finger et al.^23^ used a transcorneal approach and avoided irrigation. In our series, we kept the cyst capsular space open to the anterior aqueous humor to make it difficult to reclose. The cumulative therapy success rate was as high as 96% at 1 year and 65% at 10 years. At the final follow-up (mean, 47.4 ± 46.9 months), no EIC recurred in 89.7% (26/29) of patients.

Epithelial sheet-like ingrowth is a potentially disastrous complication following anterior segment surgery and penetrating ocular injuries. The sheet-like growth in anterior chamber structures and posteriorly over the ciliary body and even the retina can place a burden on the en bloc section. As early as in 1978, Stark et al.^24^ described 10 consecutive cases treated by photocoagulation to define the extent of epithelial involvement, followed by excision of the involved iris tissue and vitreous, and cryotherapy of the epithelium remaining on the posterior surface of the cornea, ciliary body, and in the anterior chamber. During the 23-month follow-up, eight patients achieved improved vision, two had controlled IOP with medication, and one had an IOP of 6 mmHg. In 1992, Naumann and Rummelt^18^ reported a block excision without cryotherapy in 32 consecutive patients with an average follow-up of 60.1 months, demonstrating no recurrence and visual acuity ≥ 20/60 in 37.5% of patients. The major postoperative complication was corneal endothelial decompensation in 9 patients, among whom 4 received antiglaucomatous medication. With the development of modern microsurgery, epithelial ingrowth has been uncommon. The sheet-like ingrowth in one of our patients (Fig. 3) seeded in the former operative incision for anterior chamber iris cysts was successfully treated by “open iridectomy” surgery with no recurrence observed over 4 years of follow-up. However, the sheet-like ingrowth located in the sclera progressed slowly for 20 years after the previous surgery. We performed the resection and partial allograft scleral transplantation at the area according to As-OCT images. Antiglaucomatous eyedrops were administered for 5 months to control the IOP. During the 10-month follow-up after the secondary surgery, corneal endothelial decompensation did not become aggravated, and thereby corneal transplantation was not needed.

Because of the low occurrence rate of iris cysts, this study was limited as the retrospective design. The pathological findings and influencing factors of the recurrence also need further clarifications based on more clinical cases. Nevertheless, we provided a new idea for repeatable and minimally invasive treatment with ample information of the etiology, postoperative complications, and recurrence from a comparatively large number of patients with a long-term period of follow-up. The “open iridectomy” can be effective in the treatment of secondary anterior chamber EICs, with a low rate of recurrence and favorable preservation of the corneal epithelium.

## Supplementary Information

Supplementary Video 1.
